# Association between Reactive Oxygen Species, Transcription Factors, and Candidate Genes in Drought-Resistant Sorghum

**DOI:** 10.3390/ijms25126464

**Published:** 2024-06-12

**Authors:** Jiao Liu, Xin Wang, Hao Wu, Yiming Zhu, Irshad Ahmad, Guichun Dong, Guisheng Zhou, Yanqing Wu

**Affiliations:** 1 Joint International Laboratory of Agriculture and Agri-Product Safety, Yangzhou University, Yangzhou 225000, China; jiaoliu0407@163.com (J.L.); wangxin2203@163.com (X.W.); yzuwuhao@163.com (H.W.); mx120220721@stu.yzu.edu.cn (Y.Z.); irshadgadoon737@yahoo.com (I.A.); 2 Jiangsu Key Laboratory of Crop Cultivation and Physiology, Yangzhou University, Yangzhou 225000, China; gcdong@yzu.edu.cn

**Keywords:** drought stress, sorghum, agronomic traits, reactive oxygen species, transcription factor, genes

## Abstract

Drought stress is one of the most severe natural disasters in terms of its frequency, length, impact intensity, and associated losses, making it a significant threat to agricultural productivity. Sorghum (*Sorghum bicolor*), a C4 plant, shows a wide range of morphological, physiological, and biochemical adaptations in response to drought stress, paving the way for it to endure harsh environments. In arid environments, sorghum exhibits enhanced water uptake and reduced dissipation through its morphological activity, allowing it to withstand drought stress. Sorghum exhibits physiological and biochemical resistance to drought, primarily by adjusting its osmotic potential, scavenging reactive oxygen species, and changing the activities of its antioxidant enzymes. In addition, certain sorghum genes exhibit downregulation capabilities in response to drought stress. Therefore, in the current review, we explore drought tolerance in sorghum, encompassing its morphological characteristics and physiological mechanisms and the identification and selection of its functional genes. The use of modern biotechnological and molecular biological approaches to improving sorghum resistance is critical for selecting and breeding drought-tolerant sorghum varieties.

## 1. Introduction

Drought stress, one of the primary abiotic stresses, affects plant growth and development, potentially leading to disastrous outcomes. It hinders plant metabolic activities, such as gas exchange between leaves and cells, and it causes oxidative damage, which decreases yields [1,2]. Drought stress is a multidimensional abiotic stress that affects plants at molecular, morphological, physiological, and biochemical levels [3]. Drought stress may cause photosynthesis to stop and metabolic issues to occur, which can lead to plant death.

Sorghum (*Sorghum bicolor*) is a C4 “high-energy plant” known for its exceptional growth characteristics, as well as its high stress tolerance, rapid growth rate, and high biomass production [4]. It serves as a valuable source of cereals, fodder, and sugar. Sorghum is regarded as an exemplary example of excellent plant drought tolerance due to its inherent ability to withstand drought, diploid genome structure, and efficient photosynthetic system [5]. However, sorghum, a high-quality feed crop grown around the world, has been struggling in recent years due to climate change [6]. This has had a significant impact on its growth and crop quality, particularly during droughts. As a result, to ensure long-term sorghum production, it is critical to increase the crop’s drought tolerance and investigate the processes underlying its drought resistance [7].

The main ways that sorghum can handle drought are through its agronomic traits, antioxidant defense mechanisms, ability to scavenge reactive oxygen species (ROS), and related transcription factors [8]. Drought tolerance is a polygenic trait that involves multiple genes in physio-morphological, molecular, and biochemical processes and pathways. Therefore, this review aimed to understand how drought-tolerant sorghum functions under drought stress. Section 1 emphasizes how drought affects the morphological characteristics of sorghum roots and how to mitigate those effects. Section 2 focuses on sorghum’s physiological drought response mechanisms, focusing on its ROS scavenging and antioxidant systems for maintaining its normal cellular metabolism. Section 3 involves the identification of functional genes in drought-resistant sorghum and related transcription factors. This review’s investigation will assist agronomists and breeders with developing and implementing drought-tolerant sorghum varieties capable of adjusting to changing production settings in arid locations. Integrating traditional and molecular breeding techniques with rapid generational advancement methods can reduce sorghum’s breeding cycle and improve the effectiveness of introducing new types.

## 2. Agronomic Traits of Sorghum under Drought Stress

Under drought stress, sorghum exhibits a variety of agronomic traits, including reduced plant height, leaf wilting and crumpling, decreased numbers of spikes, and deficient grain filling [9]. These modifications eventually lead to changes in its growth and phenotype, and such morphological traits are clear signs of how well different types of sorghum can handle drought, and thus, they are useful tools for identifying drought-tolerant sorghum [4]. Numerous studies have indicated close correlations between sorghum’s plant height morphology and its drought tolerance [5,10]. Under drought-tolerant conditions, sorghum biomass was significantly higher than that of drought-sensitive plants [11,12]. Emendack et al. [13] demonstrated that plant height, particularly in the early and late stages of growth, can serve as an indicator of drought tolerance in sorghum. In the early growth stages, drought-prone sorghum cultivars showed little-to-no germination, and in the later growth stages, they showed signs of male sterility, poor seed sets, and decreased yields. Bai et al. [14] showed that plant height and spike length were positively correlated with yield in sorghum under constant environmental conditions. Similarly, Zhou et al. [15] demonstrated that plant height and spike length diversity indices were greater than other agronomic characteristics in a study on sorghum germplasm resources, examining them as indicators of drought tolerance in sorghum under drought conditions. Under drought conditions, sorghum growth status not only determines the plant’s photosynthetic area, production potential, and final yield but also exerts feedback regulation on its internal metabolism. Therefore, we can identify drought resistance in crops using indicators such as the seed germination rate, survival rate, plant height, dry matter accumulation rate, leaf area, number of yellow and withered leaves, leaf expansion rate, and interval between pollen shedding and silk emergence.

Under drought stress conditions, the root system’s ability to absorb water directly affects sorghum’s drought resistance [16]. Various factors, including drought tolerance and different sorghum varieties, influence the growth and development of the root system. For example, under drought stress conditions, the root systems of drought-resistant varieties are more developed than those of non-drought-resistant varieties [17]. Additionally, the same sorghum varieties have more developed root systems under moderate drought stress conditions compared to normal growth conditions. These research findings have been confirmed by Meyer et al. [16], who showed a strong positive correlation between drought tolerance and sorghum root lengths. Habyarimana et al. [17] also sought to address this association, observing that sorghum drought tolerance was dependent on its ability to acquire water from deeper soil regions in order to maintain an appropriate water content. Similar studies have also shown that sorghum roots can keep plants from drying out. This is accomplished by increasing root biomass, root length, and root volume expansion and improving root length density (RDL) [17]. Under limited water conditions, Blum et al., found that the length of the roots directly influenced the amount of water absorbed [18]. Additionally, Stone et al. [19] demonstrated that sorghum roots exhibited a consistent penetration rate of 3.4 cm per day, reaching a plateau approximately 10 days after flowering. These studies emphasized the importance of sorghum roots extending beyond the reach of accessible water during drought circumstances, with increased root lengths allowing the plants to acquire water from deeper soil layers and ameliorate the consequences of drought stress. There are significant genetic differences in root characteristics among varieties, with high heritability for traits such as root diameter, root dry weight, root length, and root density, while the heritability of root pulling force is relatively low. Dominant, additive, and cascading genetic effects control the expression of many genes related to drought resistance, influencing the phenotypic changes in sorghum roots under drought stress [20]. Notably, a drought-tolerant sorghum line possessed roots that extended at least 40 cm deeper than a drought-sensitive one, and deeper rooting of stay-green lines under drought conditions was reported [21]. In another study, under drought stress, drought-tolerant sorghum varieties exhibited larger root–shoot ratios, longer total root lengths, and greater root surface areas, emphasizing the critical function of increased root length in conferring drought tolerance [22].

### QTL Mapping of Drought Tolerance in Sorghum Pre- and Post-Flowering under Drought Stress Conditions

Drought tolerance in sorghum is a complex quantitative trait influenced by a combination of major and minor genes, as well as genotypic and environmental factors [23]. Molecular marker technology has been extensively utilized to investigate the genetic basis of drought tolerance in sorghum, aiding in the identification and localization of QTLs associated with drought tolerance [24]. Sorghum has exhibited distinct response traits to drought stress, including pre-bloom and post-bloom drought tolerance, which may govern different genetic mechanisms. This explanation of genetic control and response traits has provided valuable insights into the molecular mechanisms of drought tolerance in sorghum [25,26,27].

Rosenow et al. [28] studied drought-tolerant sorghum hybrid cultivar selection and breeding for agricultural purposes, focusing on both the pre- and post-flowering stages. In particular, the location of the QTL for pre-flowering drought tolerance affected important yield-related traits in sorghum, such as the number of spike grains, the weight of one thousand kernels, and the overall seed yield. Tuinstra et al. [29] showed that a cross between pre-flowering drought-tolerant RTx7078 and pre-flowering drought-sensitive B35 developed a recombinant inbred line (RIL) population comprising 98 individuals. Their study identified six QTLs associated with pre-flowering drought resistance, with two QTLs in linkage group D, three in linkage group F, and one in linkage group M. Similarly, Kebede et al., used an RIL population of 125 individuals derived from a cross between pre-flowering drought-resistant SC56 and pre-flowering drought-sensitive RTx7000 [30]. Their research showed that four QTLs—Prf C, Prf E, Prf F, and Prf G—were linked to pre-flowering drought resistance. These QTLs explained 11.9% to 37.7% of the phenotypic variation. Notably, Prf E and Prf F originated from the drought-tolerant parental SC56, while Prf C and Prf G originated from the drought-sensitive parental RTx7000. These findings offer valuable insights into the genetic basis of pre-flowering drought tolerance in sorghum and provide essential information for breeding programs aimed at enhancing drought resilience in this important crop.

The localization of QTLs for drought tolerance after flowering in sorghum is critical due to the impact of post-flowering drought on plant physiology and yield. Post-flowering drought stress in sorghum leads to early leaf senescence, reduced chlorophyll content, diminished photosynthetic capacity, hindered grain filling, and, ultimately, reduced yield [31]. These effects can be primarily attributed to variations in flowering time, resulting in phenological differences. Sabadin et al., identified significant co-localization between flowering times and grain yields in sorghum, along with three QTLs, one of which was located on chromosome 9 and controlled both flowering time and plant height [32]. Their study provides valuable insights into the genetic basis of seed yields and QTLs in sorghum after flowering, with potential associations with plant height and seed yield. In another study, Sakhi et al., investigated 107 sorghum germplasm resources under drought treatments, starting at the spikelet stage [33]. Using ninety-eight pairs of SSR markers, they analyzed the association of twenty-three drought-tolerance traits and detected a total of nine QTLs associated with eight drought-tolerance traits, including stem thickness, leaf drying rate, flowering time, spikelet stipe protruding length, and flag leaf length. The identified QTLs and molecular markers can serve as a solid foundation for the precise localization of post-flowering drought-tolerance genes and the elucidation of their mechanisms of action in sorghum. These findings have contributed to a deeper understanding of the genetic basis of drought tolerance after flowering in sorghum, offering potential targets for molecular breeding strategies aimed at enhancing drought resilience in this important crop.

The integration of marker-assisted selection is pivotal in the screening and localization of QTLs for enhancing drought tolerance in sorghum lines [34]. A population was developed through pre-flowering and post-flowering backcrosses of sorghum with drought stress-sensitive lines, enabling the identification of QTLs on chromosomes controlling flowering time and plant height, thereby enhancing tolerance. The optimization of existing markers for the efficient detection of QTLs associated with drought tolerance in sorghum is crucial for advancing breeding efforts in this crop.

## 3. Mechanism of Physiological Response in Drought-Resistant Sorghum

### 3.1. Response of Photosynthetic System to Drought Stress

**Figure 1:**
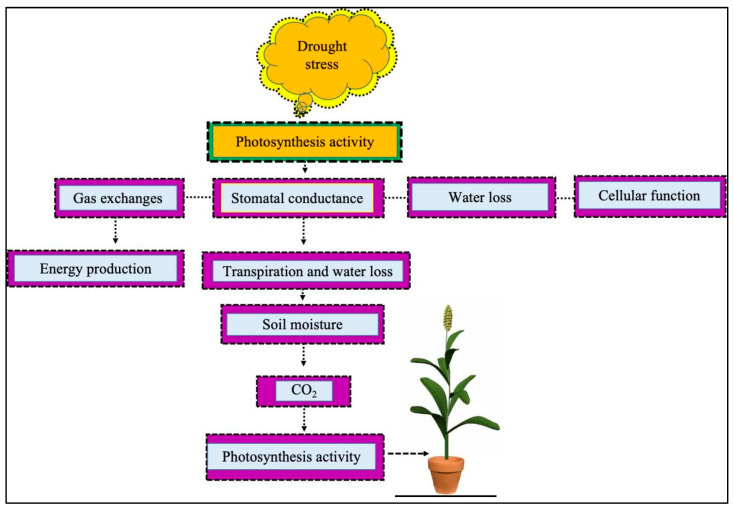
Drought stress adversely affects plant photosynthesis, and as a result, it affects gas exchange, stomatal conductance, water loss, and cellular functions. Gas exchange plays a key role in energy production. In addition, stomatal conductance regulates transpiration rates and water loss. When moisture content is limited during drought stress, it reduces the entry of CO_2_ and, as a result, decreases photosynthesis activity in sorghum.

Under drought stress, the regulation of photosynthesis in leaves is an important phenomenon in controlling plant water loss [35]. During drought stress, plants reduce photosynthesis activity as a result of increased water loss, disturbed cellular functions, stomatal conductance, and gas exchange (Figure 1). Gas exchange is a key mechanism in plant tissues for maintaining cellular functions and energy production. Stomatal regulation plays an important role in preventing transpiration and water loss through the stomata, with stomatal opening often resulting in up to 90% water loss [36,37]. When transpiration rates increase, stomatal closure reduces water loss in sorghum. The negative correlation between sorghum’s stomatal conductance and drought tolerance serves as a potential marker of drought tolerance [38]. Moreover, stomatal regulation is a key mechanism of an important cellular activity involving the maintenance of cellular water regulation [39]. Stomatal conductance (Gs) and transpiration rate (Tr) have been suggested as traits that can be used for marker-assisted selection in sorghum [40]. When soil moisture is insufficient, stomata tend to reduce transpiration rates through partial or total closure, which reduces water loss while reducing CO_2_ entry, thus leading to decreases in the photosynthetic rates (Pn). It was found that the net photosynthetic rate (Pn), photochemical efficiency (Fv/Fm), photochemical burst (qP), and actual photosystem efficiency (ΦPSII) of sorghum decreased under drought stress.

These physiological and ecological characteristics reflect sorghum seedlings’ physiological tolerance to drought stress. Is this physiological expression present in sorghum under drought stress at all reproductive stages? Can sorghum show similar physiological incentives to other crops after relieving stress? These questions require in depth study as they are important in guiding the development of appropriate field-management measures such as timely irrigation.

### 3.2. Response of Reactive Oxygen Metabolism System to Drought Stress

ROS are important signaling molecules in the cellular response to abiotic stress [41]. Drought stress induces the production of ROS such as superoxide radicals (·O_2_^−^), hydroxyl radicals (·OH), and H_2_O_2_. Previous research has shown that the ·OH in ROS directly induces the peroxidative catabolism of unsaturated fatty acid chains in phospholipids, disrupting the overall membrane. This leads to metabolic dysregulation in vivo.

#### 3.2.1. Participation in Signal Transduction

Previous research has demonstrated that ROS, as second messengers, are involved in various responses such as cell growth and development [42], programmed cell death (PCD) [43], hormone signaling [44], and different biotic and abiotic stresses by altering the redox states of cells [45].

**Figure 2:**
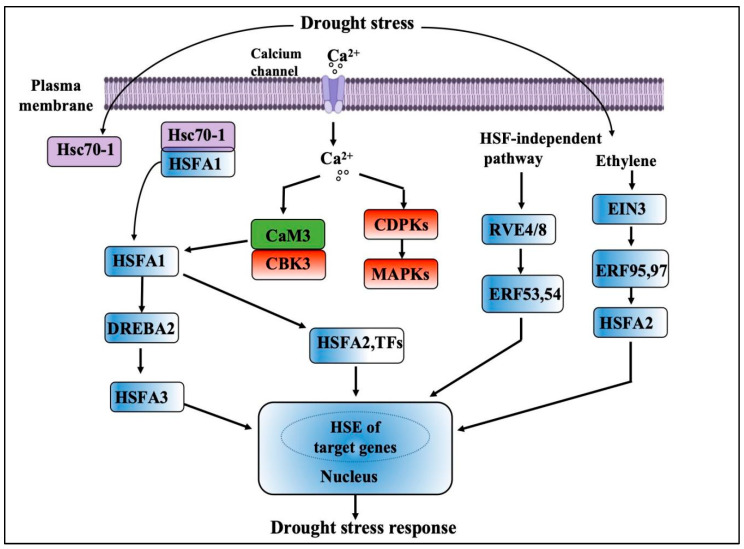
Plant ROS transcription-factor signaling network.

In recent years, the investigation of plant adversity stress signal transduction (ROS) has become an essential component of cellular signal regulation and transmission [2]. NADPH oxidase at the plasma membrane is one of the major pathways for ROS production in plants. When plants are exposed to biotic or abiotic stress, NADPH oxidase rapidly catalyzes the production of large amounts of O_2_− from O_2_. The superoxide is then converted into other ROS, such as hydrogen peroxide (H_2_O_2_), by SOD hydroxyl (OH) radicals [46]. In sorghum, NADPH generates ROS which act as signaling molecules that activate stress response pathways and enzymes and lead gene expression changes under drought stress [47]. Moreover, NADPH-mediated ROS production maintains cellular homeostasis in these plants. ROS can modulate ion-channel activities in sorghum, trigger the synthesis of osmoprotectants, and regulate cell-wall strengthening, and as a result, cell structures and functions are maintained under drought stress conditions [48]. The Ca^2+^ concentrations in plasma membranes depend on the degree of plasma membrane Ca^2+^ channel activity or opening, the degree of activation of cytosolic Ca^2+^ pumps, etc. Among the aforementioned regulatory elements, ROS demonstrate a distinct regulatory influence (Figure 2).

In turn, Ca^2+^ initiates NADPH oxidase, which induces ROS production and a greater cytoplasmic Ca^2+^ inward flow. Additionally, ROS act as second messengers in *Arabidopsis thaliana*, using HCN to break down seed dormancy [42]. ROS are also involved in signal transduction for the development of PCD in plants. Previous studies have shown that different ROS have specific functions in inducing signal transduction for PCD [43]. The mutant fluorescent (flu) produces monoclinic oxygen species in alternating light/dark environments, and the mutant stimulates a cell-death response immediately after the release of monoclinic oxygen species [49].

#### 3.2.2. Involvement in ROS Metabolism

Stress primarily affects a plant’s ROS-free-radical metabolism, the extent of lipid peroxidation in its cell membranes, and the activities of its antioxidant enzymes. There are two types of processes involved in ROS metabolism. The first are enzymatic antioxidants such as SOD, CAT, and APX, and the second are non-enzymatic antioxidants such as ASH, GSH, and alkaloids, which also remove intracellular ROS. CAT is a peroxisome signature enzyme that can convert H_2_O_2_ into O_2_ and water and plays an indispensable role in the clearance of H_2_O_2_ from peroxisomes. Also, glutathione peroxidases (GPXs), GSTs, and peroxiredoxin (PRXs) [50] lower H_2_O_2_ and organic hydroperoxides through a thiol-mediated pathway that does not depend on ascorbate [50], and they do so using an ascorbate-independent thiol-mediated pathway to decrease H_2_O_2_ and organic hydroperoxides. These pathways work together to eliminate ROS. The antioxidant enzymes such as SOD, CAT, ascorbate peroxidase (APX), NADH-dependent dehydroascorbate reductase (NADH), glutathione reductase (GR), and glutathione peroxidase (GPX), and monodehydroascorbate reductase (MDAR), APX, GR, and NADH mitigate H_2_O_2_ through the Halliwell–Asada pathway [51,52]. The two primary approaches now used to represent major antioxidant enzyme activities (e.g., SOD, CAT, and APX) are as follows: first, comparing two control groups under two different drought and water situations [53], and second, summarizing the activities of the total extracted enzymes [54]. In order to examine the impacts of drought stress on the expression and activity of the major antioxidant enzymes such as SOD, CAT, and APX in a more comprehensive manner, it is common practice to utilize antioxidant enzyme expression and activity levels across a range of drought conditions [55]. In sorghum, ROS generated by NADPH oxidase activate antioxidant defense mechanisms. These mechanisms consist of the upregulation of antioxidant enzymes such as CAT, POD, and SOD, which alleviate the adverse effects of oxidative stress caused by the excessive accumulation of ROS under drought stress [48].

Harb et al., conducted transcriptomics research to investigate the effects of moderate drought stress on *Arabidopsis* [56]. To conduct a comprehensive analysis, they identified 406 *Arabidopsis* genes that encode fundamental components of antioxidant and redox homeostasis. Although this group of genes does not represent all proteins that may be involved in ROS-related metabolism or repair, it represents many known antioxidant and reductant regeneration enzymes. Finding redox-linked genes that are sensitive to drought shows how different antioxidative and redox homeostatic systems respond. However, the response within each class is complex and unique. Most notably, there is no general upregulation of ROS-producing enzymes or antioxidative and redox homeostatic pathways. In all categories, as many genes are repressed as are induced.

In 2022, Zheng et al., overexpressed *SbNAC9* in sorghum and discovered that the transgenic lines had better photosynthesis abilities, root structures, and ROS scavenging abilities, which made the sorghum more resistant to drought [57]. *SbNAC9* can also directly turn on the putative peroxidase gene *SbC5YQ75* and the putative enzyme gene *SbNCED3*, both of which are involved in making ABA [58]. Silencing *SbC5YQ75* and *SbNCED3* via VIGS resulted in reduced drought tolerance and decreased ABA content in the sorghum seedlings.

## 4. Transcription Factors Involved in the Drought Stress Response

During drought stress signal transduction, transcription factors (TFs) play crucial roles in plant growth and development under abiotic stress by initiating multiple pathways to regulate and reduce plant stress damage at multiple levels [59]. Over 1700 family genes encoding more than 50 transcription factors have been isolated from a model *Arabidopsis* plant. Among them, the main transcription factor gene families related to drought, salt, and heavy metal stress were AP2/ERF, HD-ZIP/bZIP, NAC, MYB, C2HC, and WRKY.

### 4.1. Response of HD-ZIP Transcription Factors to Drought Stress

**Figure 3:**
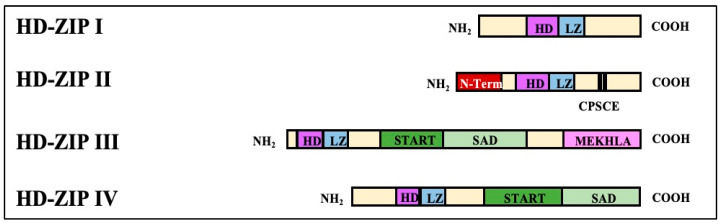
The classifications and structures of the HD-ZIP transcription factors.

HD-ZIP transcription factors are transcription factors that are only found in higher plants. They have a structure that includes a homodimeric domain (HD) that binds DNA and a zipper domain (ZIP) that interacts with proteins, with leucine at the C-terminus of the homodimeric domain [60,61]. Research has demonstrated that drought, excessive salt, ABA, and cold damage trigger genes belonging to subfamilies I and II of the HD-ZIP family of transcription factors. These two genes are involved in the hormone signaling pathway and regulate plant-cell expansion, division, and differentiation by interacting with hormone pathway genes and downstream genes, thus improving plant stress resistance [62,63,64]. Depending on the structures, conserved sequences, physiological functions, and other structural domains contained in the HD-ZIP protein genes, this family of proteins can be classified into four major classes (I–IV) [65,66], of which the first two are predominant in number (Figure 3).

The HD-ZIP transcription factors can improve the morphological characteristics of plants under abiotic stress. Sorghum is characterized by its small genome and short life cycle [67]. A relevant study indicated that, among the 1644 common differentially expressed genes (DEGs) in sorghum roots under severe drought stress and rehydration treatments, 2 HD-ZIP genes were upregulated under drought stress and downregulated under rehydration treatments while 4 HD-ZIP genes were downregulated under drought stress and upregulated under rehydration treatments. This suggested that HD-ZIP transcription factors have different expression patterns in sorghum roots [68].

Also, as a C4 crop, maize can lay the foundation for sorghum research. Using the pre-sequencing data for the maize transcriptome, a functional validation of the HD-ZIP transcription factors in maize plants found 42 genes that coded for HD-ZIP proteins [69]. The evolutionary tree analysis classified the 42 Zmhdz genes into 4 major categories, which aligned with the classification of the HD-ZIP genes in rice and *Arabidopsis*, demonstrating a high degree of sequence conservation in HD-ZIP genes. Researchers have found that overexpressing the HD-ZIP class genes *HaHB1* and *ATHB13* not only improved the drought tolerance of transgenic plants but also increased their biomass [70].

Transgenic rice that overexpressed the HD-ZIP class I gene *Zmhdz4* became more resistant to drought [71]. However, the overexpression of this gene in rice decreased its tolerance to salt stress [72], with a similar function to the *Arabidopsis thaliana* HD-ZIP class I transcription factor *ATHB6* [73,74]. In addition, it has been shown that members of the HD-ZIP class I family respond to stress through either the ABA-dependent pathway or the non-ABA-dependent pathway [75,76].

HD-ZIP II genes are mainly regulated by illumination conditions and auxin responses [77,78]. As a negative regulator, *ATHB2* can recognize its promoter region and participate in regulating the *Arabidopsis* shade-avoidance response [79]. *HAHB10*, an HD-ZIP II gene, shares a similar gene structure and expression pattern with *ATHB2*, which mainly regulates a plant’s response to light quality and quantity as related to plant development [80]. Recent research has shown that the expression of two cotton HD-ZIP II genes, *GhHB2* and -4, can be upregulated in hypocotyls, cotyledons, and roots by external GA_3_ treatments. These genes are crucial components of the phytohormone signaling system that controls early seedling development [81].

The HD-ZIP class III family of proteins contains the following five members: *ATHB88*, *CORONA (CNA)*, *PHABULOSA (PHB)*, *PHA VOLUTA (PHV)*, and *REV*. These genes are HD-ZIP class III family proteins, and increasing evidence has shown that they play major roles in embryonic development, cell differentiation, xylem formation, and lateral organ polarity transportation [82,83]. Previous studies on *Arabidopsis* have identified five HD-ZIP class III genes (*IFL1*, *ATHB8*, *ATHB9*, *ATHB14*, and *ATHB15*), and IFL1, *ATHB-9*, and *ATHB-14* were found to be connected to the development of the proximal axial regions of the apical meristematic tissues, the vascular bundles, and the lateral tissues, and they influenced the development of the root tips in the embryonic period [84]. Phytohormones and KANS transcriptionally regulate HD-ZIP III-mediated root development, while miR165/166 post-transcriptionally regulates it [85]. In addition, the overexpression of miR166G in *Arabidopsis* Jabba-1D (JBA-1D) mutant plants has been found to affect the transcription of HD-ZIP III homologous structural domain-leucine zip family genes [86]. HD-ZIP III genes all have ABRE and MBS action elements, but relevant analyses of their resistance abilities have yet to be reported, and further studies are needed to investigate their roles in response to drought stress. HD-ZIP IV genes are generally involved in determining outer cell fates [87,88]. The HD-ZIP class IV gene *AtHDG11* enhances drought tolerance in transgenic plants [89,90], and its homologous genes *Zmhdz14* and *Zmhdz13* also enhance drought tolerance in transgenic plants [69]. It has been hypothesized that genes in the same branch of the evolutionary tree have similar functions in sorghum resistance to abiotic stress [91]. Overexpression of the HD-ZIP class IV genes *Zmhdz14* and *Zmhdz13* not only enhanced the drought tolerance of transgenic plants but also their sensitivity to ABA [89]. Further, the leaf epidermis expressed OCL4, which encodes the maize HD-ZIP IV gene. *Arabidopsis* transgenic plants overexpressing OCL4 showed suppressed trichome development, while OCL4 RNAi transgenic plants showed ectopic trichome differentiation in their leaf margins [92].

Adverse hormone response elements have been detected in all 42 maize HD-ZIP genes. The ABA response elements appeared most frequently at 185, accounting for 47.07% of the total number of promoters in the study. Except for the HD-ZIP class I gene *Zmhdz41*, the HD-ZIP class II genes *Zmhdz37* and *Zmhdz14*, and the HD-ZIP class IV genes *Zmhdz35* and *Zmhdz30*, which did not contain ABA response elements, the remaining 37 (88.10% of the total number of genes) had 1 or more ABA response elements. Among the stress elements, the highest number of genes containing MBS elements was 23 (54.76% of the total number of genes), and these were shown to be positively induced by drought stress in the transcriptome data, with numbers of 10, 9, 0, and 4 in HD-ZIP I-IV, respectively. All 21 genes had MBS elements except for *Zmhdz14* (HD-ZIP II) and *Zmhdz35* (HD-ZIP IV), which did not contain ABRE elements.

**Figure 4:**
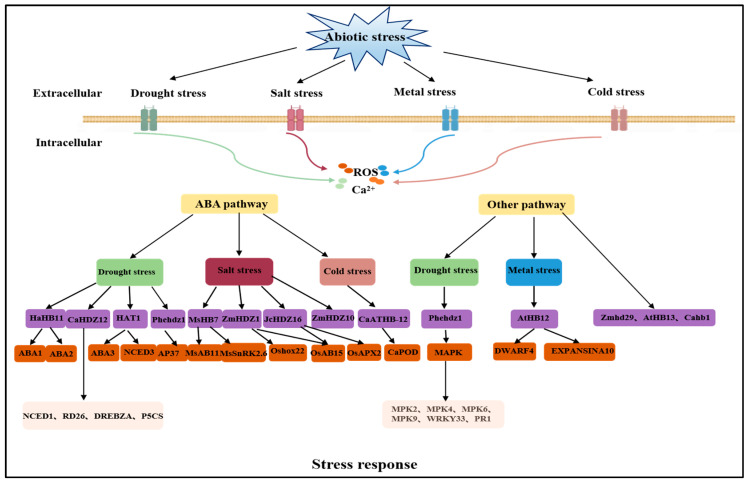
Molecular mechanisms of the HD-ZIP proteins under abiotic stress.

As a C4 crop, sorghum’s HD-ZIP transcription factors have received less attention, though maize, another C4 crop, has contributed to genetic, evolutionary, and other fundamental biological investigations. The availability of the sorghum genome sequences has provided an excellent opportunity for whole-genome annotation, classification, and comparative genomics research. Although HD-ZIP genes have been extensively characterized in *Arabidopsis* [93], rice [94], and other species [95], systemic analyses of the HD-ZIP gene family, especially for the potential stress-responsive members, have not been reported for many important species. The HD-ZIP genes in the maize genome have been found and are described at the beginning of this subsection. Furthermore, we investigated the transcript levels of HD-ZIP I genes in response to drought stress, which severely affects sorghum yields. The regulatory network of HD-ZIP proteins in the drought stress response is shown in Figure 4. The results provide a biological reference for future studies on the functions of the HD-ZIP genes and will be helpful for breeding drought-resistant sorghum.

### 4.2. Roles of the MYB Transcription Factors in Sorghum under Drought Stress

MYB functions have been extensively investigated in different plant species. An extensive review by Ambawat et al., described the roles of MYB TFs in different plant processes, including abiotic and biotic stress responses [96]. There has been a lot of research on MYB TFs; therefore, it is important to explore how they may improve stress tolerance in agricultural crops. For example, Fang et al., demonstrated that MYB TFs are active factors in abiotic stress signaling, and MYBs have been found to regulate downstream genes in response to abiotic stresses and potentially act at both the transcriptional and post-transcriptional levels [97]. Last, but not least, Baldoni et al., reviewed the roles of MYB TFs in drought response mechanisms, providing specific examples of MYB functions and discussing potential applications of MYBs [98].

Under drought stress, MYB-like transcription factors regulate cell division and differentiation, biosynthesis, stomatal development, transpiration, photosynthesis, and root development. *AMYB60* and *AMYB44* in the model crop *Arabidopsis* are transcriptional repressors that regulate guard-cell development and stomatal movement. According to Scully et al., the overexpression of *SbMYB60* promoted monophenol production in drought-tolerant sorghum [99]. The MYB TF gene has also been an area of interest in other cereals. A new gene, *OsMYB48-1*, induced ABA, hydrogen peroxide, dehydration, and PEG under salt and drought stress treatments, according to a study [100] on the role of MYB TFs in drought tolerance in rice. RNA sequencing studies have shown that transgenic maize that overexpressed OsMYB55 was better able to handle drought and high temperatures. It also turned on a lot of genes that deal with stress [101]. More recently, Tang et al., found that *OsMYB6* overexpression in rice enhanced salt and drought stress tolerance compared to wild-type plants [102]. The overexpression of *MYB15* increased the sensitivity of plants to signals such as ABA and early drying, and it caused a greater number of stomata to close. Genes such as *ABA1/2*, *ABI3*, *AiADH1*, *RD22*, *RD29B*, and *AtEM6*, which are involved in ABA production, signal transduction, and response, had increased expression at the same time. Under drought stress, the overexpression lines showed significantly better water loss and survival than the control plants at both the nutrient and reproductive stages. The presence of *MYB15* homologous genes in plants such as tobacco, tomato, barley, and wheat is important for improving the drought tolerance of these plants. The overexpression of *OSMYB4* or its homologous genes significantly improved the tolerance to drought stress in *Arabidopsis* and tomato and apple plants.

MYB transcription factors, in addition to reacting to drought stress, play essential roles in plant responses to other environmental factors such as nutrient deficiencies, salt stress, and hypoxia stress. For example, the *R_1_R_2_R_3_MYB* transcription factor gene *OsMYB3R-2*, cloned from rice, was overexpressed in *Arabidopsis thaliana*, and the transgenic plants were significantly more tolerant to freezing, drought, and high-salt stress [103]. In sorghum plants under drought stress, MYB TFs were shown to regulate genes involved in water conversation, osmotic balance, and the antioxidant defense system [68]. This included the genes that encode enzymes for osmolyte biosynthesis (proline and glycine betain). Moreover, the MYB TFs helped to minimize water loss through transpiration, thereby conserving water in sorghum plants under drought stress [68].

Although several MYB genes have been functionally characterized in model and non-model species, the characterization of these TF genes in sorghum is limited. The genome-wide identification of MYB TFs in sorghum will be critical for understanding their stress-related functions and developing improved varieties.

### 4.3. NAC Improves Reactive Oxygen Species’ Scavenging Capacities

NAC family transcription factors have numerous biological activities in different plants. So far, researchers have identified 117 NAC transcription factors in *Arabidopsis* and 151 in rice, 147 *ZmNAC* transcription factors in maize [104], and 131 NAC transcription factors in sorghum [105]. Many NAC TFs have been reported to be involved in plant responses to abiotic stress.

The *SNAC1* gene in rice was cloned in drought and high-salt conditions. The overexpression of this gene significantly improved drought and salt tolerance in rice, with a 22–34% increase in yield compared to wild-type rice [106]. Similarly, the overexpression of the *SNAC2* gene increased drought resistance in rice yields compared to wild-type rice yields [107,108]. Tran et al., cloned three *Arabidopsis* NAC family genes, *ANAC019*, *ANAC055*, and *ANAC072*, and they discovered that the overexpression of these genes dramatically increased drought tolerance compared to the wild type, with *ANAC072* being engaged in the ABA signaling system [109]. These results indicated that, as a transcription factor, *SNAC1* can actively regulate the expression of stress-related genes, and it may find application value in the improvement of drought resistance in crops.

It has been shown that the *GsNAC2* gene of the NAC family modulates sorghum’s response to salt stress via various mechanisms. A total of 20 genes associated with the ABA signaling pathway were identified as being differentially expressed in the plant hormone signaling pathway. Among these, the negative regulator SbPP2C15 was downregulated following salt-stress-induced overexpression of the *GsNAC2* gene in sorghum. The positive regulator genes *SbSAPK9* and *SbABCI12* were found to be upregulated. The GA signaling pathway enriched 19 differentially expressed genes, and following the salt–alkali stress-induced overexpression of the *GsNAC2* gene in sorghum, the receptor genes *SbChitin1* and *SbChitin2* were elevated, while the negative regulators SbSHORT-ROOT1 and SbRGL1 were downregulated. The JA signaling pathway was enriched in 23 differentially expressed genes, with the receptor genes *SbTIFY9* and *SbTIFY11e*, the positive regulator *SbJAR1*, and the negative regulator *SbbHLH61* being significantly upregulated after the salt–alkali stress-induced overexpression of the *GsNAC2* gene in sorghum. Sorghum has shown the significant upregulation of key genes *GCL*, *GS*, *GSH-Px*, and *GR* following the salt–alkali stress-induced overexpression of the *GsNAC2* gene, and *GSH-Px* and GR enzyme activities were also significantly higher than those in the control group, with 18 differentially expressed genes enriched in the glutathione metabolism pathway.

In conclusion, the *GsNAC2* gene is important to sorghum’s ability to deal with stress because it helps plants grow and develop, makes them better at getting rid of reactive oxygen species, controls glutathione metabolism pathways, and boosts the production of reduced glutathione. During drought stress conditions, sorghum accumulates proline, betainan, and soluble sugar to maintain cell turgor and protect cellular structures [8]. *GsNAC2* improves the expression of the genes involved in these osmolytes’ biosynthesis, thereby contributing to better adjustment and enhanced drought tolerance in sorghum [110]. In sorghum, the GsNAC2 genes are involved in the regulation of the antioxidant defense mechanism by upregulating the genes that encode antioxidant enzymes such as SOD, CAT, and POD, which mitigate the excessive production of ROS and protect sorghum cells from oxidative damage [111]. All of these processes improve transgenic sorghum’s stress tolerance.

To validate their RNA-Seq results, Zhang et al., utilized gene-specific primers and the qRT-PCR method to validate differentially expressed genes (DEGs) with NAC expression patterns [68]. In the leaves, five different regulatory factors in response to drought (mild and severe) and rehydration treatments were confirmed. The expression level ratios of drought and the corresponding control treatments determined by qRT-PCR were compared with the expression level ratios determined by RNA-Seq. There were significant correlations between the RNA-Seq and qRT-PCR data (r^2^ = 0.786). The qRT-PCR results showed that the expression patterns of these genes were similar to those in the RNA-Seq analysis results.

## 5. Genes and Mechanisms Related to Drought Resistance in Sorghum

The completion of the sorghum genome sequence provides an effective guarantee for the cloning of genes related to drought and stress tolerance in sorghum, despite the fact that the primary QTL for drought resistance in sorghum has not yet been available in the form of corresponding candidate genes through the mapping and cloning technique. Abdel-Ghany et al., identified a set of genes (BTx623 [DR1] and SC56 [DR2]) that exhibited differential expression only in resistant genotypes in response to PEG-induced drought [20]. This group of genes encodes known transcription factors, other proteins (signaling and metabolic enzymes) associated with drought response, and many novel proteins with no known functions. A bioinformatics analysis revealed potential regulatory elements in these genes and cognate transcription factors that may modulate their expression. To further understand seedling-stage drought tolerance in sorghum and aid in the development of drought-resistant crops, future functional investigations using the uncharacterized genes expressed only in the drought-resistant genotypes are required.

Han et al. [112] isolated and cloned the sorghum *ERECTA* gene and analyzed its sequence structure as well as its expression pattern in response to drought stress during the seedling stage. The results showed that both the *Sb ER1* and *Sb ER2* genes were expressed in sorghum stems and leaves, and their expression levels gradually increased with the severity of drought stress, and so the two genes, *Sb ER1* and *Sb ER2*, may be prime candidates for genetic engineering. Liu et al. [110] cloned the *Sb SKIP* gene from sorghum and transferred it into tobacco for a drought function analysis. The findings provided the groundwork for further studies on the role of the *Sb SKIP* gene by demonstrating that its expression enhanced the tobacco plants’ drought tolerance. In contrast, the cloning and functional analysis of the gene provided basic information for the creation of transgenic drought-resistant materials.

## 6. Conclusions

Abiotic stress severely reduces plant growth and yield. To mitigate the adverse effects of abiotic stress on sorghum, the plant uses a variety of responses, such as biochemical, physiological, and molecular responses.

Expanding China’s food production is a great strategy to ensure food security and alleviate resource deficiencies under abiotic stress, including drought stress in sorghum. However, progress has yet to be made in selecting and improving drought-resistant plants. It is still a challenge to reach the greatest yield potential due to issues with low planting efficiency and lower yields of drought-prone species in the promotion and use of their new varieties.

## 7. Future Recommendations

The current investigation provides a comprehensive review of the agronomic traits, reactive oxygen species metabolism mechanisms, and genetic basis of drought tolerance in sorghum. We presented the latest advances in sorghum morphology, physiology, and transcription factors, providing a broad understanding of these aspects. Additionally, we discussed the interactions between drought-induced ROS and the MAPK, NAC, and MYB signaling pathways. The various mechanisms that enable plant cells to withstand stress appear to be interconnected, and the degree of their interrelation is influenced by environmental factors. Due to the complexity of drought tolerance and the challenges in phenotypic aspects, the molecular genetic bases and foundations of these mechanisms have not been fully explored. Despite previous improvements, there is still tremendous potential for enhancing drought tolerance. Understanding the interplay between plant biology, cellular physiology, and plant–environment interactions requires the latest genetic information.

It has been confirmed that drought, salt, and heavy metals stresses can be alleviated via HD-ZIP transcription factor genes, such as AP2/ERF, HD-ZIP/bZIP, NAC, MYB, C2HC, and WRKY, which can be isolated from a model *Arabidopsis* plant and rice plants. However, the roles of these various genes in stressed sorghum have yet to be studied in order to alleviate the adverse effects of drought.

## Data Availability

Data used in this article are present in the tables and figures.
